# MRI Detection of Hepatic *N*-Acetylcysteine Uptake in Mice

**DOI:** 10.3390/biomedicines10092138

**Published:** 2022-08-31

**Authors:** Johnny Chen, Dennis W. Hwang, Yu-Wen Chen, Tsai-Chen Chen, Nirbhay N. Yadav, Timothy Stait-Gardner, William S. Price, Gang Zheng

**Affiliations:** 1 Nanoscale Organisation and Dynamics Group, School of Science, Western Sydney University, Penrith, NSW 2751, Australia; 2 Institute of Biomedical Sciences, Academia Sinica, Taipei 115, Taiwan; 3 Biomedical Translation Research Center, Academia Sinica, Taipei 115, Taiwan; 4 Russell H. Morgan Department of Radiology and Radiological Science, School of Medicine, The Johns Hopkins University, Baltimore, MD 21205, USA; 5 F. M. Kirby Research Center for Functional Brain Imaging, Kennedy Krieger Institute, Baltimore, MD 21205, USA

**Keywords:** *N*-acetylcysteine, glutathione, chemical exchange saturation transfer (CEST), thiol proton exchange

## Abstract

This proof-of-concept study looked at the feasibility of using a thiol–water proton exchange (i.e., CEST) MRI contrast to detect in vivo hepatic *N*-acetylcysteine (NAC) uptake. The feasibility of detecting NAC-induced glutathione (GSH) biosynthesis using CEST MRI was also investigated. The detectability of the GSH amide and NAC thiol CEST effect at *B*_0_ = 7 T was determined in phantom experiments and simulations. C57BL/6 mice were injected intravenously (IV) with 50 g L^−1^ NAC in PBS (pH 7) during MRI acquisition. The dynamic magnetisation transfer ratio (MTR) and partial Z-spectral data were generated from the acquisition of measurements of the upfield NAC thiol and downfield GSH amide CEST effects in the liver. The ^1^H-NMR spectroscopy on aqueous mouse liver extracts, post-NAC-injection, was performed to verify hepatic NAC uptake. The dynamic MTR and partial Z-spectral data revealed a significant attenuation of the mouse liver MR signal when a saturation pulse was applied at −2.7 ppm (i.e., NAC thiol proton resonance) after the IV injection of the NAC solution. The ^1^H-NMR data revealed the presence of hepatic NAC, which coincided strongly with the increased upfield MTR in the dynamic CEST data, providing strong evidence that hepatic NAC uptake was detected. However, this MTR enhancement was attributed to a combination of NAC thiol CEST and some other upfield MT-generating mechanism(s) to be identified in future studies. The detection of hepatic GSH via its amide CEST MRI contrast was inconclusive based on the current results.

## 1. Introduction

*N*-acetylcysteine (NAC) is a pharmaceutical drug with widespread applications, including the treatment of acetaminophen (paracetamol) poisoning [1,2,3,4,5], chronic obstructive pulmonary disease [6,7,8], HIV/AIDS [9,10,11,12], Alzheimer’s disease [13], diabetes [14], colon cancer [15] and cardiac dysfunction [16]. Although the therapeutic mechanisms of NAC are still an active area of research, it is generally believed that its therapeutic properties are attributed to its ability to promote the synthesis of the body’s main small-molecule antioxidant, GSH, directly scavenge harmful oxidising species and restore thiol pools to regulate the redox environment [2,17,18,19]. More importantly, it has been shown that the pharmacokinetics of NAC change in patients with chronic liver disease, resulting in elevated serum NAC concentrations and greater susceptibility to anaphylactoid reactions in NAC therapy [4,20]. Therefore, understanding the in vivo pharmacokinetics of hepatic NAC uptake on a patient level could allow for dosage tailoring in NAC therapies. Currently, the conventional method for studying the pharmacokinetic behaviour of NAC is to measure the plasma NAC concentration as a function of time, which provides limited information on the tissue-specific pharmacokinetics [4,21]. Although the high-spatial-resolution in vivo detection of NAC opens up the possibility of tissue-specific NAC pharmacokinetics in situ, which could aid in the understanding of the therapeutic effects of NAC in some of the aforementioned tissue-specific chronic diseases (e.g., Alzheimer’s disease), the current options to accomplish this would require the use of radiolabelled NAC [22], which is not as readily available for theragnostic applications.

Recently, we showed that the thiol functional group of non-radiolabelled NAC can elicit a chemical exchange saturation transfer (CEST) MRI contrast upfield from water (i.e., −2.7 ppm) at physiological conditions (i.e., near-neutral pH and 37 °C) [23]. In this proof-of-concept study, we investigated the feasibility of detecting NAC uptake in mouse liver after fasting via the upfield thiol CEST MRI contrast. ^1^H-NMR spectroscopy measurements were performed on the aqueous extracts of mouse liver to validate the hepatic NAC uptake. The feasibility of detecting hepatic glutathione (GSH) replenishment following NAC injection via amide CEST MRI contrast was also investigated.

## 2. Methods

### 2.1. Chemicals

NAC, GSH, bovine serum albumin (BSA), hydrochloric acid (HCl), sodium hydroxide (NaOH), phosphate-buffered saline (PBS), methanol (CH_3_OH), chloroform (CHCl_3_), monopotassium phosphate (KH_2_PO_4_), 3-(Trimethylsilyl)propionic-2,2,3,3-d_4_ acid sodium salt (TSP-d_4_, and sodium azide (NaN_3_) were purchased from Sigma Aldrich (Saint Louis, MO, USA).

### 2.2. Animal MRI Experiments

An NAC solution for intravenous (IV) injection was prepared by dissolving NAC crystals in 10 mM PBS to obtain a concentration of 50 g L^−1^. The solution pH was adjusted to 7 using HCl and NaOH. A control solution containing only 10 mM PBS, with the solution’s pH adjusted to 7 using HCl and NaOH, was also prepared.

All animal MRI experiments were performed on a horizontal bore 7 T Bruker Biospec scanner equipped with a 22 mm volume transmitter coil and a 2-array mouse body surface receiver coil at the Animal Imaging Facility at Academia Sinica (Taipei, Taiwan). C57BL/6 mice (male, 10–12 weeks old, 25–29 g) were fasted with water access for approximately 6 h prior to MRI scans. The C57BL/6 mice were raised and kept in cages with LIGNOCEL^®^ 3–4 S used as an adsorbent bedding material. The cages were kept in a room with a light–dark cycle of 12 h light (7 a.m.–7 p.m. local time) and 12 h dark (7 p.m.–7 a.m. local time), an ambient temperature of 22 ± 2 °C and a relative humidity of 55 ± 10%. Outside of the fasting period, the mice had free access to reverse osmosis water (containing 0.02% HCl) and a chow diet (5053-PicoLab^®^ Rodent Diet 20). During fasting, the chow was removed and only free access to water was allowed. During the MRI experiments, mice were kept warm with a water-heated bed and were anaesthetised with 0.5–2% isoflurane in air, with the breathing rate monitored using a small animal gating system (SA instruments Inc., NY, USA) and maintained in a range of 20–30 breaths/minute. A plastic sheet was wrapped firmly around the mouse body to restrict excess body movement during the scan. The tail vein was cannulated with a hand-made catheter containing 90 μL saline solution connected to a syringe containing either the 50 g L^−1^ NAC in PBS (pH 7) test solution or the PBS (pH 7) control solution.

**Figure 1:**
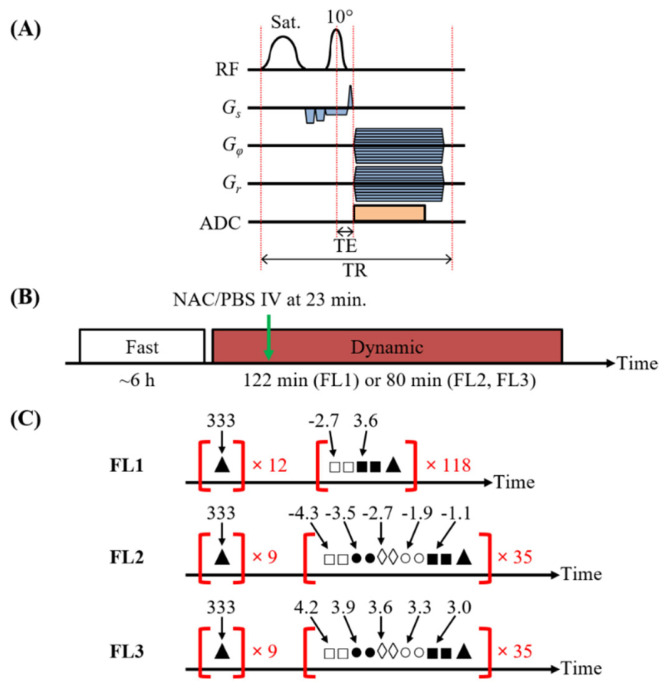
(**A**) The half-echo acquisition mode CEST-UTE pulse sequence [24]. After a 30 ms Gaussian saturation RF pulse, a crusher and pre-spoil slice-selective (G_s_) gradient was applied, followed by a 1 ms slice-selective Gaussian excitation pulse with a 10° flip angle. Subsequently, the phase-encoding (G_φ_) and readout (G_r_) gradients were switched on during the signal acquisition to acquire a single radial spoke in k-space. (**B**) The experimental procedure used in this study. In the procedure, following a fasting period to reduce liver GSH levels, a dynamic scan was performed to monitor the hepatic NAC uptake and GSH increase as a function of time. The injection (green arrow) of NAC began at 23 min into the dynamic scan. (**C**) The three different frequency lists (FL1, FL2 and FL3) used for the dynamic scan experiments. For FL1, after the 12 dummy scans with the ω_tx_ at 333 ppm, the ω_tx_ was alternated between −2.7 and 3.6 ppm. From the two iterations of the −2.7 ppm images, only the signal intensity from the second image was used in the dynamic MTR curve; the same procedure was applied to the 3.6 ppm images. For FL2 and FL3, after the 9 dummy scans, the partial Z-spectra were repeatedly acquired between −4.3 and −1.1 ppm (NAC thiol) or 4.2 and 3.0 ppm (GSH amide). Again, only the image from the second iteration of each frequency was used in the dynamic MTR curve. The interleaved 333 ppm scans were used to establish a S_0_ baseline for the dynamic MTR curve data.

Multi-slice *T*_2_-weighted images were acquired to identify an axial slice offset showing abundant liver tissue. The ultrashort echo time pulse sequence with a CEST preparation module (CEST-UTE) was used to acquire the dynamic magnetisation transfer ratio (MTR) curves (Figure 1A) [24]. A summary of the experimental protocol is shown in Figure 1B. In the CEST-UTE sequence, a 30 ms Gaussian saturation RF pulse with *B*_1_ = 1.2 μT was used, followed by a 1 ms Gaussian excitation RF pulse with a 10° flip angle to acquire a single radial spoke in k-space. Whilst a large saturation pulse amplitude (*B*_1_ > 1.2 μT) would have increased saturation efficiency, the deleterious effects of direct water saturation (DWS) and conventional magnetisation transfer (MT) at high saturation amplitudes resulted in *B*_1_ = 1.2 μT being chosen for this study. A half-echo acquisition mode was used, requiring 302 centre-out radial spokes to fill k-space, which resulted in a total saturation time for a single frequency offset of 30 ms × 302 = 9.06 s [24]. Other sequence parameters included: TR = 40.41 ms; TE = 0.66 ms; nominal Cartesian matrix size = 96 × 96; image resolution = 0.34 × 0.34 mm^2^; slice thickness = 1 mm; NA = 1; acquisition bandwidth = 100 kHz; number of trajectory sampling points = 54; and a slice-selection gradient ramp time = 110 μs. Two dynamic data sets were acquired: one where MR images with the saturation applied at −2.7 and 3.6 ppm were monitored during the scan (FL1 in Figure 1C), and one where MR images with the saturation applied in two frequency regions centred at −2.7 and 3.6 ppm (to obtain partial Z-spectra), respectively, were monitored during the scan (FL2 and FL3 in Figure 1C). For FL1, 12 dummy *S*_0_ images (i.e., MR images acquired with saturation *ω_tx_* = 333 ppm) were used to bring the system to a steady-state, followed by images acquired with the saturation *ω_tx_* alternating between −2.7 and 3.6 ppm, with additional *S*_0_ images interleaved. Two instances of the −2.7 ppm image and two instances of the 3.6 ppm image were acquired, with the first instance discarded in the final dynamic MTR curve due to a violation in the saturation steady-state from frequency switching (Figure S1 in Supplementary Materials) [24]. For FL2 and FL3, nine dummy *S*_0_ images were used to bring the system to a steady-state, followed by images acquired with the saturation *ω_tx_* swept either between −4.3 and −1.1 ppm or 4.2 and 3.0 ppm, with additional *S*_0_ images interleaved between the acquisitions of the partial Z-spectra. Again, two instances of the image at each frequency were acquired, with the first instance discarded in the final dynamic MTR curve due to a violation in the saturation steady-state from frequency switching. The resulting time resolution in the final time curve was 24.4 s for all three frequency lists. A 240 μL bolus containing the 90 μL saline solution in the catheter tube followed by 150 μL of either the 50 g L^−1^ NAC solution or 10 mM PBS solution from the syringe was injected intravenously over 1 min. The IV injection rate (240 μL min^−1^) was controlled using an automated syringe pump (Harvard Apparatus Holliston, MA, USA). The total duration of the dynamic scan was approximately 122 and 80 min for FL1 and FL2/FL3, respectively.

Pre- and post-dynamic scan (FL1) Z-spectra were acquired with the CEST-UTE pulse sequence to generate *B*_0_-shift maps. The acquisition parameters were the same as in the IV injection scans, but with the saturation *ω_tx_* being swept ±5 ppm with a frequency resolution of 0.2 ppm.

### 2.3. Preparation of Aqueous Liver Tissue Extracts for NMR Spectroscopy

In the NMR analysis of the aqueous mouse liver extracts, a test group (*n* = 6) and a control group (*n* = 6) were used. The preparation of the extracts was performed according to the procedure reported by Beckonert et al. [25]. For the test group, the tail vein of each mouse was cannulated with a hand-made catheter containing 90 μL saline solution connected to a syringe containing 50 g L^−1^ NAC in PBS (pH 7) buffer. A 240 μL bolus containing the 90 μL saline solution in the catheter tube followed by 150 μL of 50 g L^−1^ NAC solution from the syringe was injected intravenously over 1 min using an automated syringe pump (Harvard Apparatus, Holliston, MA, USA). Each mouse was sacrificed at approximately eight minutes after starting the IV injection, and a piece of the liver was immediately removed and placed in a 2 mL Eppendorf tube, weighed and then submerged in liquid N_2_. The frozen tissue was initially homogenised with 4 mL CH_3_OH and 0.85 mL H_2_O per gram of tissue, then vortexed with 2 mL g^−1^ CHCl_3_. After the addition of another 2 mL g^−1^ CHCl_3_ and 2 mL g^−1^ H_2_O, the tissue sample was vortexed again and then place in an ice-bath for 15 min. The sample was then centrifuged (1000× *g*) at 4 °C for 15 min or until the aqueous CH_3_OH/H_2_O phase was separated from the hydrophilic CHCl_3_ phase. The aqueous phase was transferred into a glass vial for solvent removal via a speed vacuum concentrator. Afterwards, the solid aqueous extracts were resuspended using 0.58 mL of a buffer solution containing 185 mM KH_2_PO_4_ + 0.71 mM TSP-d_4_ + 2 mM NaN_3_ in D_2_O. The sample was vortexed then centrifuged (12,000× *g*) for 5 min, before having 0.55 mL of the supernatant pipetted into a 5 mm NMR tube. The same IV injection protocol and liver tissue extraction procedure were used for the control group with each IV-injected bolus containing 90 μL saline solution from the catheter tube and 150 μL of a 10 mM PBS (pH 7) solution from a syringe.

### 2.4. NMR Experiments

All ^1^H-NMR experiments were performed on a Bruker Avance III HD 600 MHz WB spectrometer (Karlsruhe, Germany) using a BBFO probe equipped with a *z*-axis field gradient at the NMR Core Facility at Academia Sinica (Taipei, Taiwan). The probe temperature was calibrated to 25 °C using methanol [26]. The ^1^H-NMR spectra of aqueous mouse liver extracts were acquired using a 90° pulse-and-acquire sequence. The acquisition parameters used included: number of scans = 32; number of dummy scans = 8; receiver gain = 1; spectral width = 10 kHz; acquisition time = 2 s; relaxation/recovery delay = 20 s; FID data points = 39,998; and 90° RF pulse duration = 18.9 µs. The FIDs were apodised using an exponential window function prior to Fourier transformation.

### 2.5. Data Analysis

All data were processed using custom Python scripts. For the dynamic MTR curve obtained with all three frequency lists, the signal intensities from an ROI (i.e., liver tissue) in the *S*_0_ images were smoothed using the Savitzky–Golay filter (window size = 3, polynomial order = 2) in the SciPy Python package [27]. The resulting smoothed curve was used as a baseline, *S*_0,*SG*_, for which all other signal intensities (i.e., from the non-*S*_0_ images) were normalised against

(1) $$Z=\frac{S}{S_{0,SG}}.$$

This was performed to compensate for the signal drift associated with the *B*_0_ drift caused by the heating from the recurring application of gradient and RF pulses in the CEST-UTE pulse sequence [24,28]. Next, a pre-injection baseline correction was applied by subtracting each time-dependent *Z* data point from the average of the pre-injection *Z* data points, *Z_pre,avg_*, yielding the correct MTR value for the final dynamic MTR curve

(2) $$\mathrm{MTR}=Z_{pre,avg}-Z.$$

To generate the area-under-curve (AUC) maps, the same procedure was applied pixel-wise to obtain a dynamic MTR curve for each pixel, wherein the areas under the curve within specific time windows were calculated.

For the average dynamic MTR curve (i.e., dynamic MTR*_Avg_* curve), all MTR values from the control and test groups were averaged. The standard deviation of the data at each time point was used as the statistical uncertainty. The smoothed versions of the dynamic MTR/MTR*_Avg_* curves were generated for clarity using the Savitzky–Golay filter (window size = 7; polynomial order = 2) in the SciPy Python package.

The *B*_0_-shift maps were generated by calculating the Z-spectrum for each pixel and fitting an inverted Lorentzian to the DWS peak to estimate the RF transmitter offset value, which resulted in the smallest MR signal intensity (i.e., directly saturated water signal). This offset value, with respect to the water resonance, corresponded to the *B*_0_ shift for that pixel.

## 3. Results

### 3.1. Detection of Hepatic NAC Uptake with Dynamic Thiol CEST

**Figure 2:**
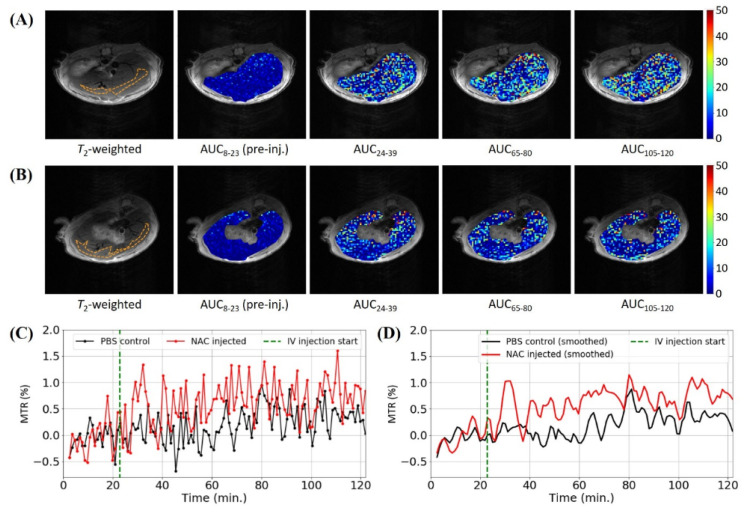
Dynamic CEST data of the representative test (i.e., NAC-injected) (**A**) and control (i.e., PBS-injected) (**B**) mice obtained through saturating at −2.7 ppm using the CEST-UTE pulse sequence. (**A**) The *T*_2_-weighted transverse slice of the mouse which was injected (IV) with NAC with its liver overlaid with multiple AUC maps. (**B**) The *T*_2_-weighted transverse slice of the mouse which was injected (IV) with PBS (control) with its liver overlaid with multiple AUC maps. The orange, dashed outline in first T_2_-weighted images in (**A**,**B**) represent the ROIs selected for plotting the dynamic MTR curves (**C**). (**D**) The smoothed version of the dynamic MTR curves in (**C**) using the Savitzky–Golay filter. The green dashed line in (**C**) represents the start of the IV injection.

In Figure 2A, the AUC maps of a representative test mouse liver were registered onto their *T*_2_-weighted anatomical MR images to show the changes in the spatial distribution of the MTR across the time windows 8–23 (AUC_8–23_) (pre-injection), 24–39 (AUC_24–39_), 65–80 (AUC_65–80_) and 105–120 (AUC_105–120_) minutes. These time windows were selected to highlight the contrast between the AUC maps obtained on the test and control mice across several regions of interest on the dynamic MTR curves. The AUC_24–39_, AUC_65–80_ and AUC_105–120_ maps showed increases in the MTR over the entire liver region compared to the pre-injection AUC_8–23_ map, suggesting the detection of the NAC thiol CEST effect in the liver. The hepatic distribution of NAC was observed to be uniform in the previous literature [22]. For comparison, Figure 2B shows the AUC maps for the same time windows of a representative control mouse liver after an IV injection of 10 mM PBS (pH 7), revealing a significantly lower MTR increase compared to the test mouse. The small but apparent MTR increase in the control post-injection AUC maps was likely a motion artefact, since restless motion in prolonged scans has been known to generate a hyper-intense pseudo-CEST contrast [29,30]. Furthermore, some of the hyper-intensity around the bottom edge of some of the post-injection AUC maps could also be attributed to chemical shift artefacts from proximal adipose tissue [31]. The average MTR magnitudes within the ROIs selected in the *T*_2_-weighted images in Figure 2A,B (orange, dashed lines) are shown as functions of the scan time (i.e., dynamic MTR curves) in Figure 2C, with the test mouse showing an increased MTR post-injection compared to the control. These ROIs were selected as to avoid any hepatic arteries/veins so that only the hepatic tissue uptake was detected. Two interesting features should be noted in the dynamic MTR curve of the test mouse: the initial MTR peak between 24 and 39 min with a maximum MTR value of 1.3% at ~32 min (i.e., ~8 min post-injection) and the gradually consistent MTR increase after 40 min (i.e., 16 min post-injection). These two features are more visible in the smoothed dynamic MTR curves in Figure 2D. Since all other CEST effects (i.e., hydroxyl, amide and amine) occurred downfield from the water resonance, the MTR increase in the test mouse dynamic MTR curve could only be explained by the upfield CEST effect of the NAC thiol group or some other unknown phenomenon.

**Figure 3:**
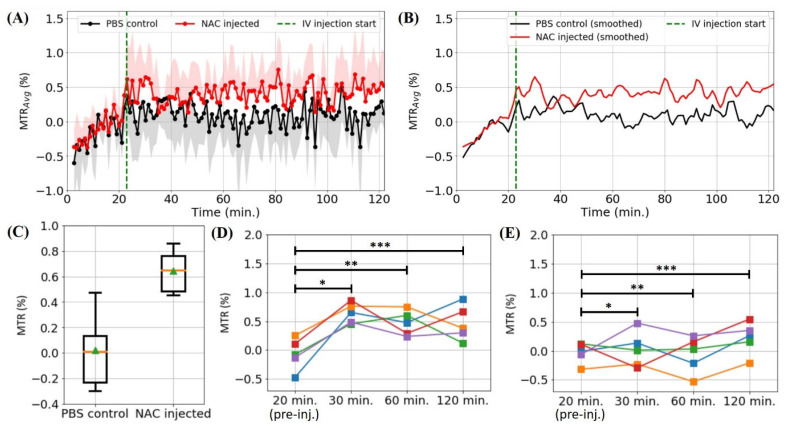
(**A**) The dynamic MTR_Avg_ curves, with the shaded regions representing the upper and lower standard deviations for the test and control group, respectively, obtained through saturating at −2.7 ppm (NAC thiol proton resonance) for the test (*n* = 5) and control (*n* = 5) mouse groups. (**B**) The smoothed version of the dynamic MTR_Avg_ curves in (**A**) using the Savitzky–Golay filter. (**C**) A box-whisker plot of the test and control group MTR values at 30 min (i.e., 6 min post-injection) (Welch’s *t*-test: *, *p* = 0.007). The boxes, whiskers, orange lines and green triangles represent the first quartiles of the data, the data extremities, the median MTR values and the average MTR values, respectively. (**D**) A comparison between the MTR values of the test group at several time points (Student’s paired *t*-test: *, *p* = 0.003; **, *p* = 0.015; ***, *p* = 0.073). (**E**) A comparison between the MTR values of the control group at several time points (Student’s paired *t*-test: *, *p* = 0.757; **, *p* = 0.787; ***, *p* = 0.034).

In the dynamic MTR*_Avg_* curve of the test group (Figure 3A,B), the two aforementioned dynamic MTR curve features were also apparent, providing stronger evidence that the hepatic NAC uptake signal enhancement was immediate, and that NAC was being accumulated. A maximum MTR*_Avg_* peak value of (0.64 ± 0.18)% was observed at ~30 min (i.e., ~6 min post-injection). In Figure 3C, the MTR values of the test group at 30 min (i.e., 6 min Spost-injection) are shown to be significantly different to those of the control group (Welch’s *t*-test, *p* = 0.007). Furthermore, Figure 3D shows that the MTR values of each test mouse measured at 30, 60 and 120 min increased with respect to the MTR value measured at 20 min (i.e., pre-injection), with the MTR*_Avg_* value at 30 (Student’s paired *t*-test, *p* = 0.003) and 60 (Student’s paired *t*-test, *p* = 0.015) minutes being significantly different to the MTR*_Avg_* value at 20 min. The MTR*_Avg_* value at 120 min did not differ significantly to that at 20 min according to the standard significance level of 0.05 due to the MTR values at 120 min of two of the test mice (green and orange data sets in Figure 3D) being smaller than the MTR values measured at 60 min. In comparison, the control MTR*_Avg_* values at the 30 and 60 min did not differ significantly from the pre-injection MTR*_Avg_* value (Student’s paired *t*-test, *p* > 0.05 for both), whereas the control MTR*_Avg_* value at 120 min was statistically different to the pre-injection MTR*_Avg_* value (Student’s paired *t*-test, *p* = 0.034) (possibly due to the motion-induced pseudo-CEST artifact increasing the MTR*_Avg_*).

**Figure 4:**
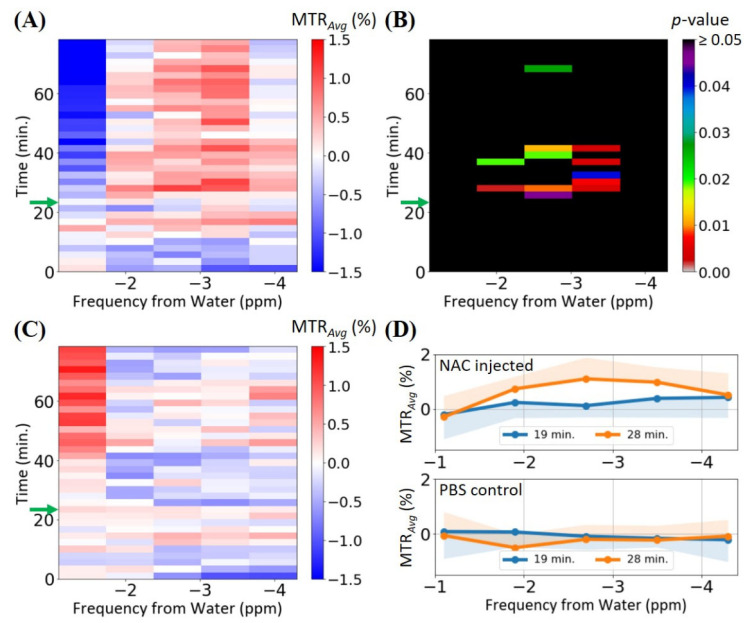
The partial Z-spectral (−4.3–−1.1 ppm) data for the test (*n* = 5) and control (*n* = 5) mouse groups. (**A**) A 2D map showing the MTR_Avg_ magnitudes for the test group as a function of chemical shift from water and experimental time. (**B**) A 2D map showing the experimental time and chemical shift regions where the test group’s MTR_Avg_ values were statistically different from the control group’s MTR_Avg_ values with *p* < 0.05 (Welch’s *t*-test). (**C**) A 2D map showing the MTR_Avg_ magnitudes for the control group as a function of chemical shift from water and experimental time. (**D**) The MTR_Avg_ values of the partial Z-spectra at 19 min (i.e., pre-injection) and 28 min (i.e., post-injection) for the test and control groups, with the shaded regions representing the lower and upper standard deviations for the partial Z-spectra obtained at 19 and 28 min, respectively. The height of each cell in (**A**–**C**) represents the experimental time required to acquire a single partial Z-spectrum. The five cells in each row in (**A**–**C**) correspond to the five discrete chemical shift values of the partial Z-spectra. The green arrows in (**A**–**C**) represent the start of the IV injection.

Dynamic partial Z-spectra around the NAC thiol proton resonance were acquired and averaged to observe the shape of the MTR*_Avg_* profile (Figure 4) and determine the significance of increased DWS on the MR signal attenuation from *T*_2,*w*_ broadening due to an additional *T*_2_ exchange from NAC. For each *ω_tx_* in the averaged partial Z-spectra of the test (i.e., NAC-injected) and control (i.e., PBS-injected) mouse groups, MTR*_Avg_* values were calculated and plotted as a function of time—these are shown in the 2D maps in Figure 4A and Figure 4C, respectively. Another 2D map (Figure 4B) elucidated the corresponding *p*-values less than 0.05 from the Welch’s *t*-test on the two data sets. It can be stated that a hyper-intense MTR*_Avg_* region occurred between 28 and 32.5 min (i.e., 4–8.5 min post-injection) in the test map, which corresponded to the same initial hepatic NAC uptake seen in Figure 3A. This was not observed in the control map and was found to be statistically significant. In fact, when comparing the MTR*_Avg_* profiles at 28 min with the pre-injection (19 min) MTR*_Avg_* profiles for both the test and control groups (Figure 4D), it could be seen that the post-injection MTR*_Avg_* profile for the test group was a broad peak with a maximum at −2.7 ppm. This strongly resembled the broad MTR profiles seen in the phantom experiments and simulations (Figure S2, Supplementary Materials), and did not show a significantly increased DWS, which would typically manifest as a hyper-intense region in the ±1 ppm region of the MTR profile (i.e., such as that observed in the MTR profiles in Figure S2C in the Supplementary Materials), suggesting that the MR signal attenuation between 24 and 35 min primarily comprised of the NAC thiol MTR. After 35 min, a consistent MTR*_Avg_* increase was observed in the test group, similar to that observed in Figure 3A. However, only some of these MTR*_Avg_* values between 35 and 45 min were significant, as shown by the *p*-value map, whereas the subsequent MTR*_Avg_* values were likely contaminated by the same motion-induced pseudo-CEST seen in the control data. The negative MTR*_Avg_* values in the beginning of the dynamic scans (0–10 min) were attributed to the varying MR signal intensities as the system still approached an approximate saturation steady-state. This was also observed in dynamic CEST-UTE experiments by Zhou et al. [32], where the initial averaged data point in the dynamic plot showed a much larger variance.

The effects of the *B*_0_ drift on the MR signal can be seen in Figure S3 in the Supplementary Materials. Although the *S*_0,*SG*_ baseline normalisation was used to compensate for the signal drift associated with the *B*_0_ drift (Figure S3A), the shifting (Figure S3B) and *T*_2_ broadening of the water resonance could not be accounted for using this approach.

### 3.2. Validation of Hepatic NAC Uptake by NMR Spectroscopy

**Figure 5:**
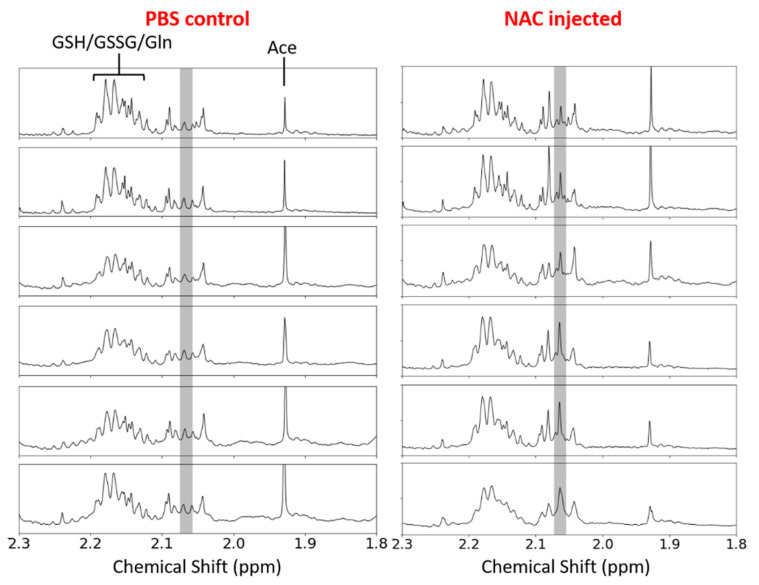
A close-up (1.8–2.3 ppm) of the ^1^H-NMR spectra of aqueous mouse liver extracts from the test (*n* = 6) and control (*n* = 6) groups to show the presence and absence of the distinct NAC methyl proton singlet at ~2.06 ppm (shaded) in the test and control groups, respectively. The resonances of GSH, GSSG, glutamine (Gln) and acetate (Ace) were also identified for reference [34,35].

To verify that the MTR increases in the test group data in Figure 2, Figure 3 and Figure 4 were due to NAC uptake, ^1^H-NMR spectral analysis was performed on the aqueous liver extracts from a test (*n* = 6) and control (*n* = 6) group. Liver tissues were extracted from the mice in both the test and control groups at ~8 min after an IV injection of 50 g L^−1^ NAC in PBS (pH 7) (test) and 10 mM PBS (pH 7) (control) solutions, respectively. Close-up ^1^H-NMR spectra of the aqueous liver extracts from the mice in each group are shown in Figure 5, with the shaded region indicating the chemical shift region of the NAC methyl proton peak [23,33]. There was a distinct singlet peak in this region in the test spectra, which could not be observed in the control spectra, strongly indicating that the hepatic NAC uptake had occurred in the test mice at ~8 min post-injection. This coincided well with the MTR/MTR*_Avg_* spike observed at ~31 min (i.e., ~7 min post-injection) in the dynamic MTR/MTR*_Avg_* curves for the test mice in Figure 2 and Figure 3, providing strong evidence that this upfield MTR increase was indeed due to the presence of an NAC thiol CEST.

## 4. Discussion

Phantom experiments and simulations were performed to determine the detectability of the NAC thiol and GSH amide CEST effects at *B*_0_ = 7 T (Section S2, Supplementary Materials). The experimental and simulated MTR profiles (Figure S2, Supplementary Materials) showed that it should be feasible for the NAC thiol and GSH amide CEST effects to be detected at 37 °C and at *B*_0_ = 7 T.

The liver was chosen as the organ of interest in the study of the NAC uptake, since it is the most important metabolic site for pharmaceutical drugs. The dynamic NAC CEST data were obtained by repeatedly saturating the NAC thiol proton resonance frequency (i.e., −2.7 ppm from water), during which NAC or PBS solutions were injected at 23 min into the CEST-UTE experiments. A summary of the dynamic CEST data for the representative test and control mice is shown in Figure 2. The initial MTR spike was likely due to the immediate hepatic uptake of NAC, which would be consistent with the immediate increase in mouse liver tissue radioactivity upon the IV injection of ^14^C-labelled NAC, as demonstrated by McLellan et al. [22]. The subsequent MTR increase possibly indicated the accumulation of NAC in the hepatic tissue but may also have been overestimated due to the presence of a motion-induced pseudo-CEST artifact at longer scan times, which was also observed in the control mouse data. These two MTR features, the initial spike and subsequent gradual increase, were recurring features in the test group, as shown by the test MTR*_Avg_* curve (Figure 3A). However, the NAC-enhanced MTR signal of some of the test mice appeared to depreciate, as shown in Figure 3D. It was difficult to establish the exact cause for this decrease, since the magnitude of the CEST effect is, in general, complex, due to its dependence on multiple factors (e.g., CEST CA concentration, proton exchange rate, **B_0_** homogeneity and *T*_1,*w*_). McLellan et al., observed decreasing ^14^C radioactivity after ~45 min after the post-injection of ^14^C-labelled NAC, possibly due to a decreasing hepatic NAC concentration—this may explain the decreasing NAC thiol MTR at longer scan times. Decreases in *T*_1,*w*_ and *T*_2,*w*_ values are also known to decrease CEST effects (Section S7, Supplementary Materials) [36], but this was not likely to be a significant factor in this study, as this would imply a large change in the paramagnetic species (e.g., hepatic iron) concentration. To the best of the authors’ knowledge, there was no evidence of NAC uptake modulating the endogenous hepatic iron concentration.

Although the dynamic MTR/MTR*_Avg_* curves (i.e., Figure 2C and Figure 3A) when saturating the NAC thiol proton resonance appeared to indicate the hepatic uptake of NAC, it was uncertain if the increased DWS from the water linewidth broadening due to an additional *T*_2_-exchange from NAC contributed to the MR signal attenuation. The acquisition of dynamic partial Z-spectra and, therefore, dynamic partial MTR*_Avg_* profiles between −4.3 and −1.1 ppm could provide some clarification. The partial MTR*_Avg_* profile acquired at 28 min (i.e., coinciding with the MTR spike at ~30 min in Figure 3A) was a broad peak with a maximum MTR*_Avg_* at −2.7 ppm. This was consistent with the simulated NAC phantom MTR profile (Figure S2C, Supplementary Materials), and showed that the MR signal attenuation was predominantly from the NAC thiol chemical exchange. Another interesting feature was the increasing hypo-intensity and hyper-intensity of the MTR*_Avg_* at −1.1 ppm in the test and control dynamic partial MTR*_Avg_* profiles, respectively. Although the hypo-intensity in the test map may have been in part due to the fast exchanging NAC thiol protons reducing the water linewidth (i.e., *T*_2_ relaxation enhancement) [37], this was not a very significant effect in the test data, as indicated by the *p*-values > 0.05 in the corresponding cells in Figure 4B. Instead, the hypo-/hyper-intensity was likely an artefact resulting from the increasing *B*_0_ drift from the recurring RF pulse and pulsed field gradient switching in the CEST-UTE pulse sequence, which would cause the shifting and broadening of the water resonance [24,28]. The degree of this shifting and broadening would vary between different mice, creating a large data variance for the MTR*_Avg_* values at −1.1 ppm. This large data variance was evident when considering that the hypo-intense and hyper-intense MTR*_Avg_* values at −1.1 ppm were not statistically significant (Figure 4B). In addition, post-processing *B*_0_-drift correction techniques, such as WASSR [38], could not be easily applied to dynamic CEST data. Nevertheless, the broad MTR profile of the NAC thiol CEST effect allowed for it to be less sensitive to such *B*_0_ inhomogeneity, particularly since this kind of field drift is usually on the order of several Hz [28].

The CEST MRI detection of GSH was also investigated (Section S4, Supplementary Materials), since GSH replenishment has been associated with hepatic NAC uptake [2,17,19,39,40]. The non-invasive detection of GSH using CEST MRI would also be beneficial in other medical diagnoses, since alterations in endogenous GSH levels have been associated with many disorders, including Parkinson’s disease [41,42], HIV [43,44,45], liver disease [4,46,47] and cystic fibrosis [48,49]. It was also shown previously that the amide–water proton exchange of GSH could generate a CEST effect at physiological conditions [23,50]. The mice were fasted since fasting is known to reduce hepatic GSH levels, which should, in theory, have allowed for a more pronounced and detectable change in GSH amide CEST contrast after NAC intake [51]. Unlike the NAC thiol MTR, the apparent GSH amide MTR was more convoluted due to the overlapping of its CEST effect with that from other endogenous labile protons (e.g., amides/amines from endogenous proteins/peptides). Although there was an apparent initial MTR/MTR*_Avg_* spike with a subsequent MTR/MTR*_Avg_* increase when saturating at 3.6 ppm (i.e., GSH amide proton resonance) in Figure S4C and Figure S5A (Supplementary Materials), the corresponding dynamic partial MTR*_Avg_* profiles also showed a similar MTR*_Avg_* increase at 3.3 ppm. This suggested that the increased CEST effects from other endogenous amides/amines, which also had their proton resonances in the same region [50,52,53,54], were generated upon the injection of the NAC solution. However, it should be noted that it was difficult to establish the true significance of these MTR*_Avg_* increases, since only a small number (*n* = 3) of experimental repetitions were performed due to time constraints. The MTR*_Avg_* increase at 3.6 ppm suggested a possible increase in the GSH amide MTR. However, previous studies have shown that significant hepatic GSH replenishment occurs only after a NAC dosage of almost four times that used in this study [55,56]. The fasting of the mice in this study was expected to reduce hepatic GSH levels by up to 10% [51], so that the post-NAC-injection GSH replenishment would be more pronounced. The effect of NAC on the hydroxyl/amide/amine CEST effects of hepatic proteins/peptides was outside the scope of this study, and remains the subject of future investigations.

To verify that the initial upfield MTR/MTR*_Avg_* spike when saturating the NAC thiol proton resonance was indeed due to the presence of NAC in the mouse liver, a ^1^H-NMR spectral analysis was performed on the aqueous liver extracts from the test and control mice (Figure 5). Whilst the presence of a singlet peak at ~2.06 ppm in the ^1^H-NMR spectra of the test group provided strong indication of a hepatic NAC uptake, the spectral deconvolution quantification of the ^1^H-NMR spectra revealed an average hepatic NAC concentration of 0.12 ± 0.03 mM at ~8 min post-injection (Section S8, Supplementary Materials). Such a small hepatic NAC concentration should not have been able to elicit a detectable thiol CEST from NAC, suggesting the presence of some other upfield MT-generating mechanism. One possible explanation was the increased concentration of the intermediate product in the GSH synthesis pathway, L-γ-glutamylcysteine, a thiol-containing molecule, which could have elicited a stronger thiol CEST contrast—further investigation of the significance of this mechanism post-injection of NAC is needed. It is also entirely possible that the metabolomic NMR procedure [25] used in the liver tissue extraction may have caused the loss and/or degradation of some of the hepatic NAC, resulting in an underestimated in vivo hepatic NAC concentration—other procedures (e.g., UPLC-MS [57]) should be used for the future for a comparison of the quantified hepatic NAC concentrations.

Nevertheless, the ^1^H-NMR spectral analysis of the aqueous liver extracts did reveal the hepatic NAC uptake in the mice group injected with 50 g L^−1^ NAC in PBS (pH 7) via the distinct NAC methyl proton singlet peak at ~2.06 ppm. Whilst the small hepatic NAC concentration derived from these results may not have provided strong evidence that the post-injection upfield MTR/MTR*_Avg_* spikes observed in the test group’s dynamic MTR/MTR*_Avg_* curves between 24 and 39 min (Figure 2, Figure 3 and Figure 4) were solely due to the NAC thiol CEST contrast mechanism, they definitely suggested the enhancement of the MTR signal through a combination of an NAC thiol CEST and some other unknown mechanism(s)—much like the dynamic glucose-enhanced MRI contrast [58], which primarily attributed the glucose MTR enhancement to both a CEST and relaxation mechanism. Due to this, the direct quantification of the in vivo NAC concentration with CEST MRI was challenging, and remains a focus in future studies, as the potential clinical applications for this could include the development of biomarkers for measuring the NAC theragnostic efficacy (e.g., comparing serum and hepatic NAC concentrations to look at the uptake). The post-injection downfield MTR/MTR*_Avg_* increase in the test group’s dynamic MTR/MTR*_Avg_* curves (Figures S2–S4, Supplementary Materials) could not have been verified due to the replenishing of GSH, since no evidence of increased GSH levels could be found in the ^1^H-NMR spectral analysis. This did not discard the fact that hepatic GSH replenished after the NAC IV injection, since it is well-known that NAC increases GSH synthesis [2,5,12,22,55,59]. Rather, the non-labile GSH proton resonances were scalar-coupled, making it difficult to detect changes in their signal intensities. The ^1^H-NMR spectral analysis of aqueous mouse liver extracts for post-injection time points after ~8 min could not be performed due to time constraints and remains a subject for future investigations.

## 5. Conclusions

In conclusion, the detection of hepatic NAC uptake in mice using MRI was shown to be feasible. The upfield dynamic MTR/MTR*_Avg_* curves showed an initial MTR/MTR*_Avg_* spike with a maximum at ~7 min after the start of the IV injection of 50 g L^−1^ NAC in PBS (pH 7). The hepatic NAC uptake was verified by the presence of the NAC methyl singlet peak at ~2.06 ppm in the ^1^H-NMR spectra of aqueous mouse liver extracts obtained ~8 min after the injection of 50 g L^−1^ NAC in PBS (pH 7); however, the estimated in vivo hepatic NAC concentration, 0.12 ± 0.03 mM, from the spectral deconvolution was too low to generate a detectable CEST effect. These results suggested that the MTR/MTR*_Avg_* spike was due to an apparent combination of an NAC thiol CEST effect and other MT-generating mechanism(s), possibly as a result of NAC metabolism—this should be investigated in a future study. The MTR/MTR*_Avg_* increases observed after 17 min post-injection could not be verified as being due to an NAC accumulation in the test mouse liver tissues based on the current results. The downfield MTR/MTR*_Avg_* increase in the dynamic MTR/MTR*_Avg_* curves obtained from the test mice could not be verified as being due to the GSH replenishment from the hepatic NAC uptake because changes in the ^1^H-NMR signal intensities of the GSH non-labile protons could not be detected.

## Data Availability

Data is contained within the article and Supplementary Materials.

## Supplementary Materials

The following supporting information can be downloaded at: https://www.mdpi.com/article/10.3390/biomedicines10092138/s1**,** Figure S1: in vitro CEST MRI results for NAC and GSH, Figure S2: dynamic CEST data of the representative test and control mouse obtained through saturating at 3.6 ppm using the CEST-UTE pulse sequence, Figure S3: dynamic MTR*_Avg_* curves obtained through saturating at 3.6 ppm (i.e., GSH amide proton resonance) for the test (*n* = 5) and control (*n* = 5) mouse groups, Figure S4: the partial z-spectral (4.2–3.0 ppm) data for the test (*n* = 3) and control (*n* = 3) mouse groups, Figure S5: comparison of motion artifact compensation in UTE and RARE MR images, Figure S6: BM-simulated CD MTR profiles of 20 mM NAC and GSH at various *T*_1,s_ and *T*_2,s_ values, Figure S7: evidence of S_0_ signal drift in the ROI of a representative test mouse, Figure S8: steady-state violation in CEST-UTE experiments, Figure S9: simulated NAC thiol MTR values at different *T*_1,w_ and *T*_2,w_ values. References [60,61,62,63,64,65,66,67,68,69,70,71,72,73] are cited in the Supplementary Materials.
