# Supplementary material for: MRI Detection of Hepatic N-Acetylcysteine Uptake in Mice
Source: Biomedicines. 2022 Aug 31;10(9):2138. doi: 10.3390/biomedicines10092138 (PMC9495914; doi:10.3390/biomedicines10092138)
Supplement: Supplementary file 1 [file biomedicines-10-02138-s001.zip › biomedicines-1819277-supplementary.pdf]

Supplementary Material

MRI Detection of Hepatic *N*-Acetylcysteine Uptake in Mice

Johnny Chen,[1] Dennis W. Hwang,[2]* Yu-Wen Chen,[3] Tsai-Chen Chen,[2] Nirbhay N. Yadav,[4,5] Timothy Stait-Gardner,[1] William S. Price,[1] and Gang Zheng[1]*

[1]Nanoscale Organisation and Dynamics Group, School of Science, Western Sydney University, Penrith, NSW 2751, Australia

[2]Institute of Biomedical Sciences, Academia Sinica, Taipei, Taiwan

[3]Biomedical Translation Research Center, Academic Sinica, Taipei, Taiwan

[4]Russell H. Morgan Department of Radiology and Radiological Science, The Johns Hopkins University School of Medicine, Baltimore, MD, USA

[5]F. M. Kirby Research Center for Functional Brain Imaging, Kennedy Krieger Institute, Baltimore, MD, USA

*Correspondence to: Gang Zheng, Ph.D., Nanoscale Organisation and Dynamics Group, School of Science, Western Sydney University, Penrith, NSW 2751, Australia, e-mail: G.Zheng@westernsydney.edu.au; Dennis W Hwang, Ph.D., Institute of Biomedical Sciences, Academia Sinica, Taipei, Taiwan, e-mail: dwhwang@ibms.sinica.edu.tw

Table of Contents

S1. Phantom Experiments and Bloch-McConnell Simulations 2

S2. Detection of Hepatic GSH by Dynamic Amide CEST 5

S3. UTE vs. RARE Acquisition for Motion Artifact Compensation 9

S4. $T_{1,s}$ and $T_{2,s}$ Effects on NAC Thiol and GSH Amide MTR Profiles 11

S5. Effect of B_0 Drift on MR Signal 12

S6. Steady-state Violation in CEST-UTE Experiments 13

S7. $T_{1,w}$ and $T_{2,w}$ Effects on NAC Thiol MTR 14

S8. Hepatic NAC Concentration from ^{1}H-NMR Spectral Deconvolution 14

References 16

S1. Phantom Experiments and Bloch-McConnell Simulations

PBS phantoms of NAC and GSH were prepared by dissolving NAC and GSH crystals in separate vials containing 10 mM PBS to make a final concentration of 20 mM. The pHs of the PBS phantoms were adjusted to 7.2 using HCl and NaOH, before being transferred to 5 mm NMR tubes. BSA phantoms of NAC and GSH were also prepared by adding NAC and GSH crystals to separate vials containing BSA crystals, to which 10 mM PBS was added to each vial to make final concentrations of 20 mM NAC + 50 g L^{-1} BSA and 20 mM GSH + 50 g L^{-1} BSA. The pHs of the BSA phantoms were adjusted to 7.2 with HCl and NaOH, before being transferred to 5 mm NMR tubes. Solutions of 10 mM PBS and 50 g L^{-1} BSA in PBS, with pHs adjusted to 7.2 using HCl and NaOH, were also prepared as phantom controls. All phantom MRI experiments were performed on a horizontal bore 7 T Bruker Biospec scanner equipped with a 22 mm volume transmitter coil and a 4-array mice brain surface receiver coil. The six NMR tubes were held together with adhesive tape and placed flat on the animal bed, with a portion of the tubes covered by the receiver coil. The phantom imaging experiments were performed at room temperature. A T_2-weighted image was acquired to identify a suitable axial slice offset for the CEST MRI acquisition. The rapid acquisition with relaxation enhancement pulse sequence with a continuous-wave (CW) saturation preparation module (CEST-RARE) was used to acquire z-spectral images (1-3). A rectangular pulse with an RF amplitude (B_1) = 2 μT and duration (t_{sat}) = 4 s was used for CW saturation. The saturation RF transmitter (ω_{tx}) was swept ±5 ppm around the water resonance with a frequency resolution of 0.1 ppm to obtain the z-spectral data. Other pulse sequence parameters include: repetition time (TR) = 12 s, effective echo time (TE) = 2.87 ms, RARE factor = 3, partial Fourier acceleration factor = 1.4, matrix size = 128 × 128, image resolution = 0.34 × 0.34 mm^2, number of averages (NA) = 1, and slice thickness = 5 mm. The duration required to acquire an image slice at a single ω_{tx} was approximately six minutes.

All z-spectra simulations in this study were performed using custom Python scripts. The simulated z-spectra of 20 mM NAC (pH 7.2) and GSH (pH 7.1) at static field strength (B_0) = 7 T and 37 °C were generated by numerically solving Bloch-McConnell (BM) equations using the parameters: proton exchange rates (k_{sw}) = 6180 (NAC thiol) (4), 8 × 10^6 (GSH thiol), 85300 (GSH amine) (4), and 371 (GSH amide) s^{-1}; chemical shift with respect to water ($\Delta\omega_{sw}$) = −2.7 (NAC thiol) (4), −2.5 (GSH thiol) (4), 3.2 (GSH amine) (4), and 3.6 (GSH amide) ppm; t_{sat} = 4 s; B_1 = 2 μT; solute T_1 ($T_{1,s}$) = 1 s; solute T_2 ($T_{2,s}$) = 0.2 s; water T_1 ($T_{1,w}$) = 995 ms; water T_2 ($T_{2,w}$) = 23 ms (i.e., literature mouse liver T_1 and T_2 values at B_0 = 7 T) (5). The GSH thiol and amide k_{sw} values, and GSH amide $\Delta\omega_{sw}$ value were obtained from previous BM fitting

(unpublished). The $T_{1,s}$ and $T_{2,s}$ values were chosen based on previous literature values (4,6) and do not significantly affect the MTR (Figure S2, Supplementary Material).

The z-spectrum of each phantom was generated by plotting the normalised average MR signal intensity inside a ROI of each tube as a function of the transmitter frequency offset from water. The normalisation was done by dividing the MR signal of the ROI, S, by the average of all the signals from the same ROI in the initial 15 S_0 images, $S_{0,avg}$. The MTR values of NAC/GSH were calculated using a control difference (CD) approach, whereby the z-spectra with NAC/GSH were subtracted from the z-spectra without NAC/GSH (e.g., z-spectrum of 20 mM NAC in PBS (pH 7.2) subtracted from the z-spectrum of 10 mM PBS (pH 7.2)).

In Figure S1B, both the NAC thiol (purple, dashed box) and GSH amide (green, dashed box) were found to be very much detectable at 7 T and at room temperature, with the NAC thiol CEST effect indicating the thiol-water proton exchange is in the fast-exchange regime due to the broad line shape of its MTR profiles and the GSH amide CEST effect indicating the amide-water proton exchange is in the slow-exchange regime due to the narrow line shape of its MTR profiles (Figure S1C). The chemical shifts with the greatest MTR values for the NAC thiol and GSH amide CEST are approximately −2.7 and 3.6 ppm from water, respectively, which are consistent with previous results (4).

In order to emulate the MTR profiles at 37 °C and B_0 = 7 T (Figure S1C), the CD MTR profiles were simulated using the BM equations with BM fitted parameters from 20 mM NAC (pH 7.2) and GSH (pH 7.1) solutions at 37 °C and B_0 = 14.1 T (4). In the simulated MTR profile of the 20 mM NAC solution, a small MTR value of ~0.5% was observed at −2.7 ppm, whereas an MTR value of ~5.3% was observed at 3.6 ppm for the 20 mM GSH solution.

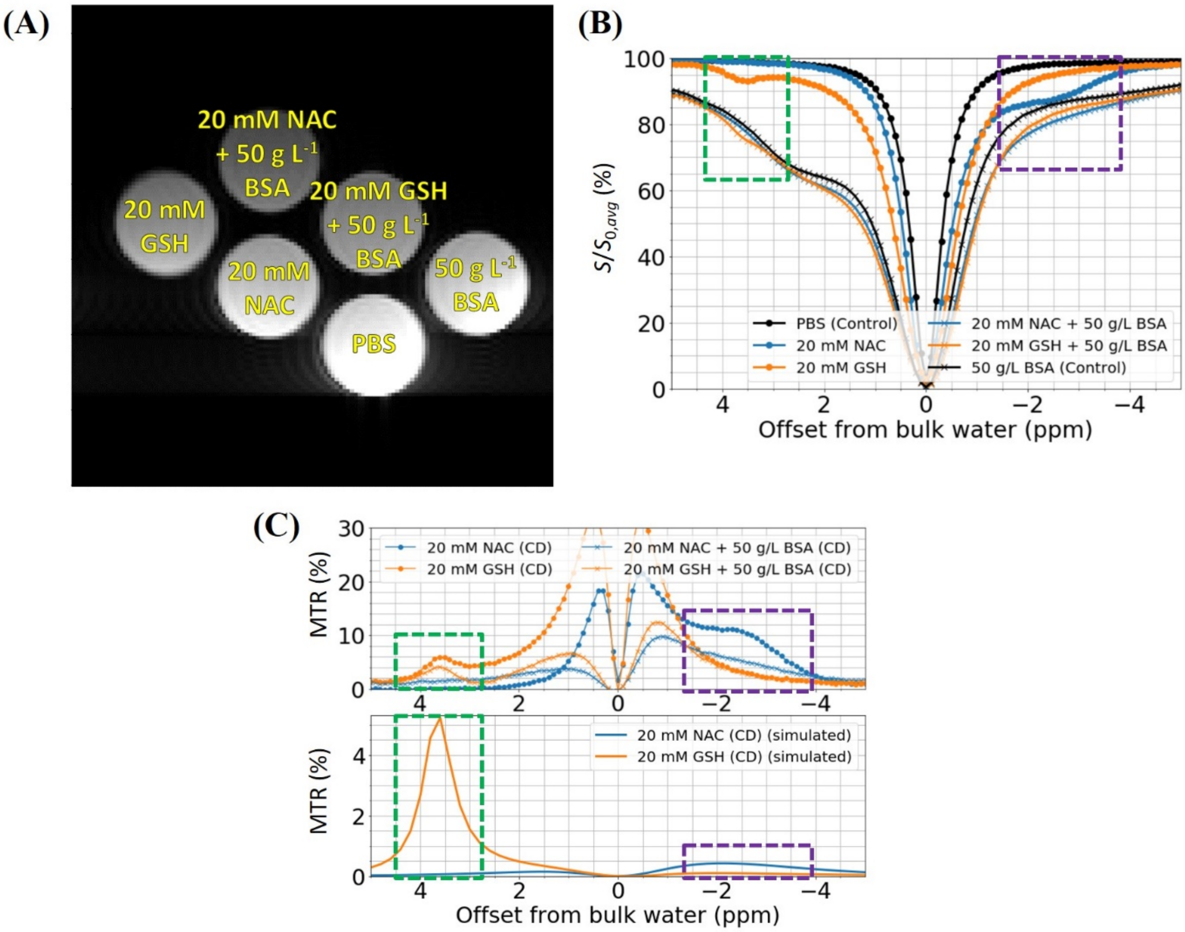

Figure S1 (A) An S_0 image showing the four test and two control phantoms: 20 mM NAC in PBS, 20 mM GSH in PBS, 20 mM NAC + 50 g L^{-1} BSA in PBS, 20 mM GSH + 50 g L^{-1} BSA in PBS, 50 g L^{-1} BSA in PBS (control), and 10 mM PBS (control). (B) The z-spectra of the phantoms. (C, top) The MTR profiles of the NAC/GSH and NAC/GSH + BSA phantoms, which were obtained by subtracting the z-spectra of the PBS and BSA test phantoms from the PBS and BSA control phantoms, respectively (i.e., control difference, or CD). (C, bottom) The BM simulated CD MTR profiles of 20 mM NAC and GSH. The MR signal gradient in (A) is due to the increasing distance between the samples and the receiver coil, which is closest to the PBS phantom.

S2. Detection of Hepatic GSH by Dynamic Amide CEST

In Figure S2, it can be seen that the post-injection AUC maps of the representative test mouse liver (Figure S2A) when saturating at 3.6 ppm (i.e., GSH amide proton) shows greater intensity compared to the representative control mouse liver (Figure S2B). This is evident in the dynamic MTR curves of the selected liver ROIs (Figure S2C and Figure S2D), where higher MTR values are observed in the test mouse compared to the control mouse. Furthermore, an initial MTR spike with a maximum at ~30 minutes and a subsequent consistent MTR increase is observed in the dynamic MTR curve of the test mouse, similar to that seen in Figure 2C and Figure 2D in the main manuscript.

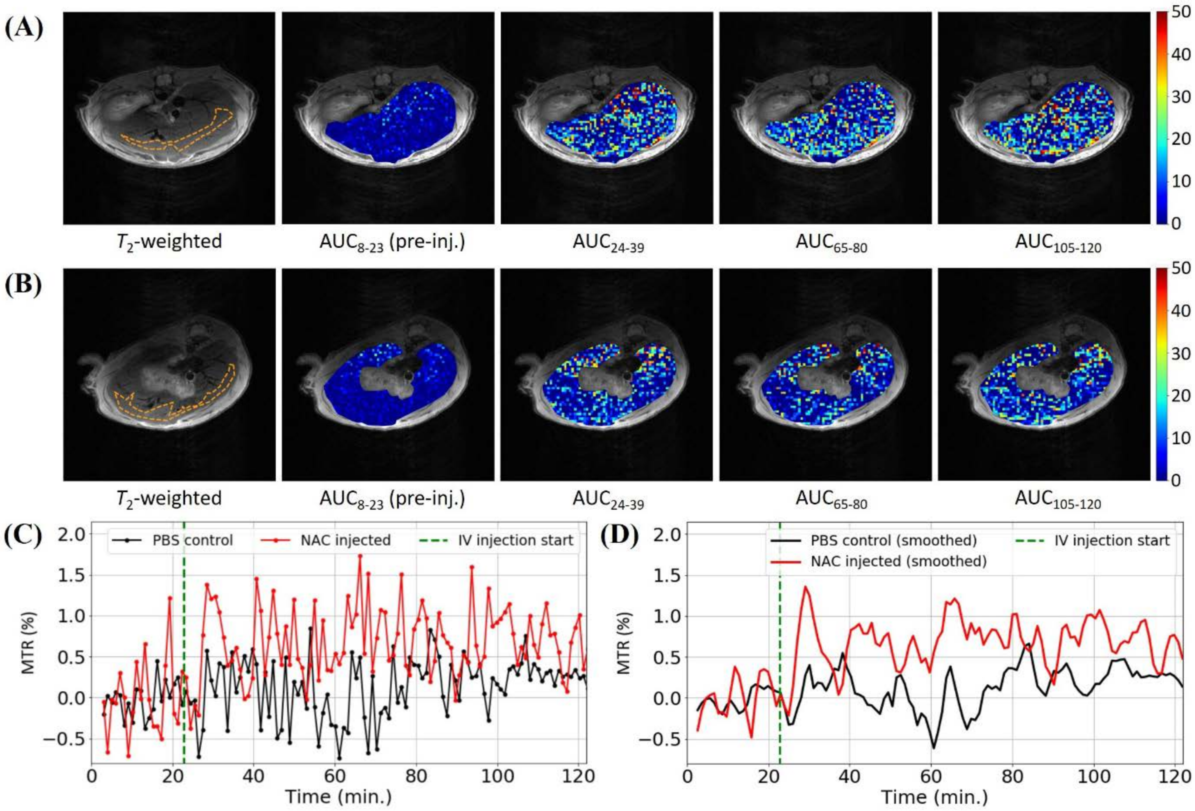

Figure S2 Dynamic CEST data of the representative test (i.e., NAC injected) and control (i.e., PBS injected) mouse obtained by saturating at 3.6 ppm using the CEST-UTE pulse sequence. (A) The T_2-weighted transverse slice of the mouse which was injected (IV) with NAC, with its liver overlaid with multiple AUC maps. (B) The T_2-weighted transverse slice of the mouse which was injected (IV) with PBS, with its liver overlaid with multiple AUC maps. The orange, dashed outlines in first T_2-weighted images in (A) and (B) represent the ROIs selected for plotting the dynamic MTR curves (C). (D) The smoothed version of the dynamic MTR curves in (C) using the Savitsky-Golay filter. The green, dashed line in (C) represents the start of the IV injection.

The dynamic MTR$_{Avg}$ curve of the test group (Figure S3A) only showed an apparent marginal MTR$_{Avg}$ increase over that of the control group. In fact, the initial MTR$_{Avg}$ spike with a maximum at ~30 minutes (i.e., ~6 minutes post-injection of NAC) in the test group was found to be insignificant compared to the control group (Welch's *t*-test, P = 0.380) (Figure S3B). When comparing the MTR values of all the test group mice obtained at 30, 60, and 120 minutes with those obtained at 20 minutes (i.e., pre-injection) (Figure S3C), it can be seen that, although the post-injection MTR values have a general increase, the changes are not as significant as observed for the NAC thiol MTR values in Figure 3D (main manuscript). Similar results were observed when comparing the post-injection MTR values of all the control group mice with the pre-injection MTR values (Figure S3D).

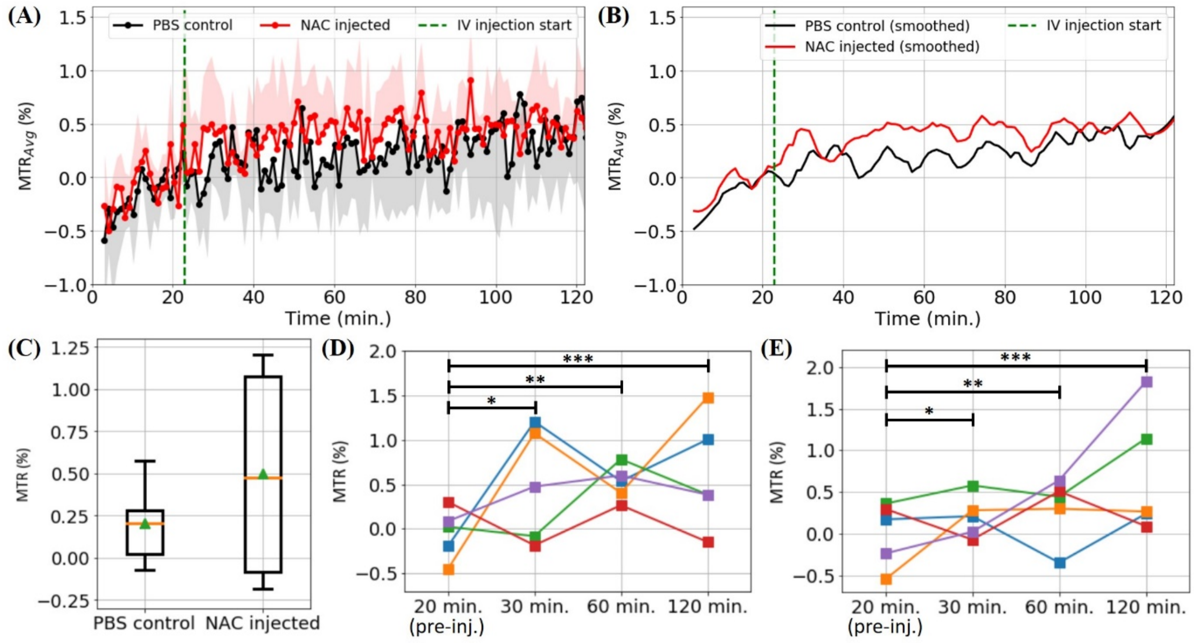

Figure S3 (A) The dynamic MTR$_{Avg}$ curves, with the shaded regions representing the upper and lower standard deviations in the test and control group, respectively, obtained by saturating at 3.6 ppm (i.e., GSH amide proton resonance) for the test (n = 5) and control (n = 5) mice groups. (B) The smoothed version of the dynamic MTR$_{Avg}$ curves in (A) using the Savitsky-Golay filter. (C) A box-whisker plot of the test and control group MTR values at 30 minutes (i.e., six minute post-injection) (Welch's *t*-test: *, P = 0.380). The boxes, whiskers, orange lines, and green triangles represent the first quartiles of the data, the data extremities, the median MTR values, and the average MTR values, respectively. (D) A comparison between the MTR values of the test group at several time points (Student's paired *t*-test: *, P = 0.246; **, P = 0.024; ***, P = 0.178). (E) A comparison between the MTR values of the control group at several time points (Student's paired *t*-test: *, P = 0.369; **, P = 0.148; ***, P = 0.148).

The averaged dynamic partial z-spectra around the GSH amide proton chemical shift for the test and control mice groups are represented as 2D maps in Figure S4A and Figure S4B, respectively. It can be seen in Figure S4A that there is a broad MTR_{Avg} increase throughout the entire partial z-spectra after IV injection of 50 g L^{-1} NAC. The pre- and post-injection MTR_{Avg} profiles (obtained 19 and 28 minutes, respectively) for both the test and control groups show clear differences, with the post-injection MTR_{Avg} profile of the test group showing very similar increases at 3.3 and 3.6 ppm in comparison with the corresponding pre-injection MTR_{Avg} profile.

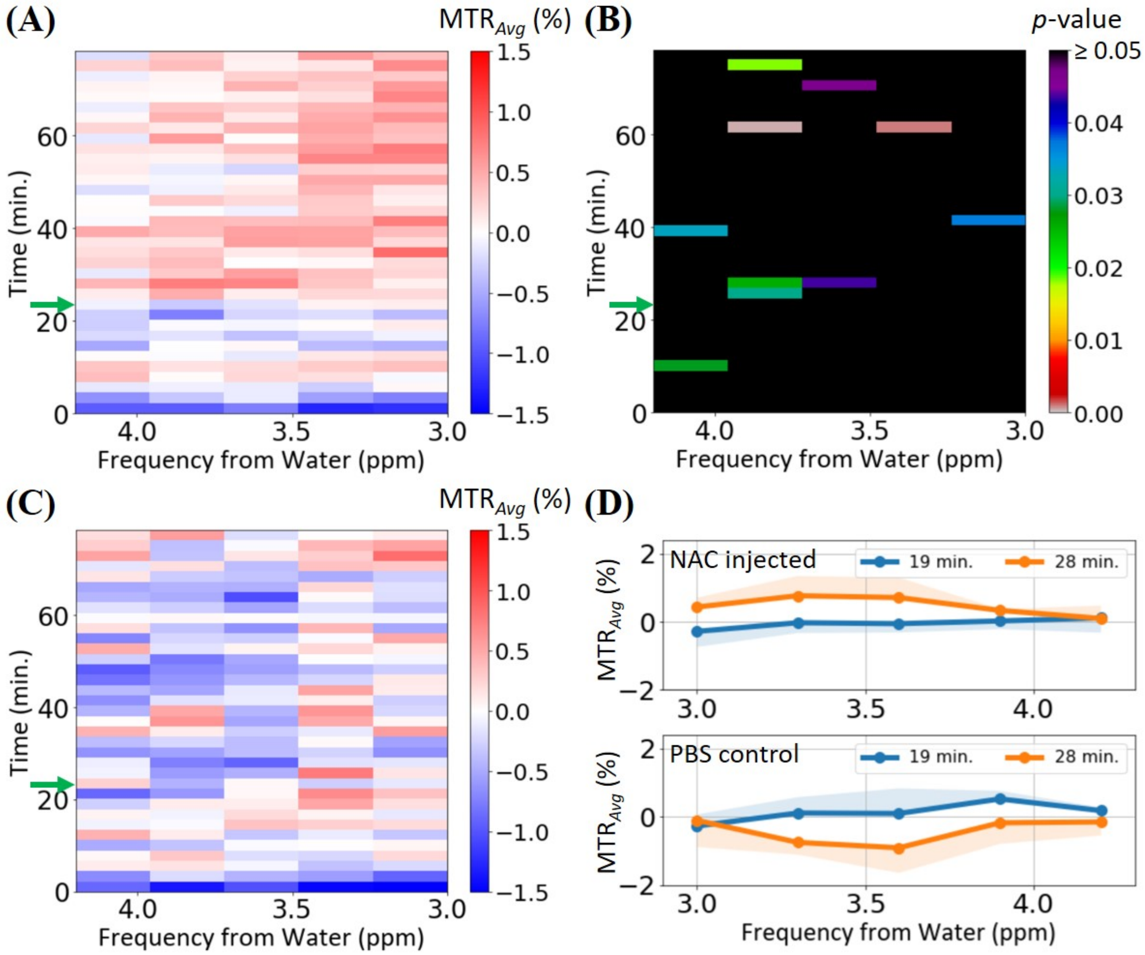

Figure S4 The partial z-spectral (4.2 – 3.0 ppm) data for the test (n = 3) and control (n = 3) mice groups. (A) A 2D map showing the MTR_{Avg} magnitudes for the test group as a function of chemical shift from water and experimental time. (B) A 2D map showing the experimental time and chemical shift regions where the test group's MTR_{Avg} values are statistically different from the control group's MTR_{Avg} values with $P < 0.05$ (Welch's *t*-test). (C) A 2D map showing the MTR_{Avg} magnitudes for the control group as a function of chemical shift from water and experimental time. (D) The MTR_{Avg} values of the partial z-spectra at 19 minutes (i.e., pre-injection) and 28 minutes (i.e., post-injection) for the test and control groups, with the shaded regions representing either the lower or upper standard deviations for the partial z-spectra obtained at 19 and 28 minutes. The height of each cell in (A), (B), and (C) represents the

experimental time required to acquire a single partial z-spectrum. The five cells in each row in (A), (B), and (C) correspond to the five discrete chemical shift values of the partial z-spectra. The green arrows in (A), (B), and (C) represent the start of the IV injection.

S3. UTE vs. RARE Acquisition for Motion Artifact Compensation

Due to the vicinity of the liver to the lungs in both mice and humans, performing MRI on the liver in vivo can be challenging due to the generation of severe artifacts in the MR images by breathing. In theory, the motion artifacts can be alleviated with the use of radial (i.e., like that used in UTE) rather than Cartesian (i.e., like that used in RARE) k-space acquisition. In Cartesian k-space, the phase encoded data are acquired over a duration on the order of tens of milliseconds to seconds (i.e., the duration required to fill the entire k-space), making the phase encoding direction of the MR image more susceptible to spin density changes caused by motion (7). Although the duration required to fill an entire radial k-space is also still on the order of tens of milliseconds to seconds, each phase encoding step occurs on the order of hundreds of microseconds to milliseconds. Due to the oversampling of the k-space centre, any radial k-space 'spokes' affected by motion is partially compensated by vicinal spokes. This causes the motion artifacts in the MR image to appear as diffused streak patterns rather than the ghost-like motion artifacts observed in Cartesian k-space reconstruction (8).

To compare the efficacy of CEST-UTE and CEST-RARE in reducing motion artifacts in the dynamic scan data, additional dynamic scans of approximately 40 minutes in duration were performed using the CEST-UTE and CEST-RARE pulse sequences with a saturation $B_1 = 0\ \mu$T. In the CEST-UTE experiment, the acquisition parameters used were the same as that used in the IV injection experiments. In the CEST-RARE experiment, the acquisition parameters used include TR = 12.2 s, effective TE = 2.52 ms, RARE factor = 23, partial Fourier acceleration factor = 1.4, matrix size = 32 × 128, image resolution = 0.23 × 0.56 mm^2, NA = 1, and slice thickness = 1 mm. For post-processing, each signal intensity was normalised against a quadratic polynomial function fitted to the data set so that the relative change in signal intensity could be observed (i.e., $S/S_{0,quad}$, where $S_{0,quad}$ is the baseline signal intensity calculated from the fitted quadratic polynomial).

In Figure S5, it can be seen that the average MR signal intensities within the mouse liver ROI obtained using the RARE acquisition fluctuate significantly over time due to severe motion artifacts, with the ROI signal intensity reduced by almost half at some time points. In contrast, the average MR signal intensities within the same ROI obtained using the UTE acquisition do not fluctuate significantly, which is important in achieving more precise dynamic MTR measurements. Thus, performing in vivo CEST MRI on liver with the CEST-UTE pulse sequence is preferable due to its robustness against motion artifacts. However, one disadvantage of using the CEST-UTE pulse sequence is that the steady-state pulsed saturation scheme results in reduced saturation efficiency compared to the CW saturation scheme used in

the CEST-RARE pulse sequence (9-11). Nevertheless, the loss of saturation efficiency in the CEST-UTE pulse sequence is justified by its significant improvement in motion compensation.

(A) (B)

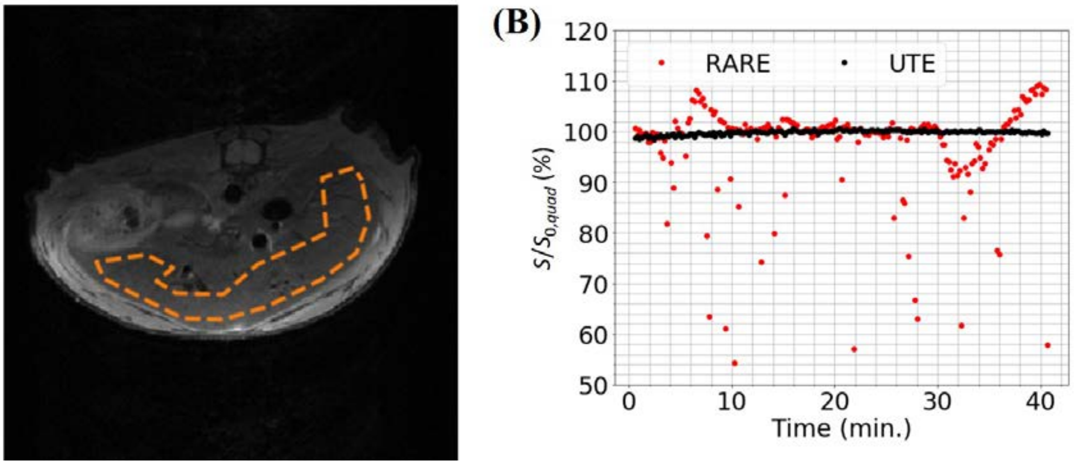

Figure S5 (A) A transverse MRI slice of a mouse showing its liver and an ROI (orange, dashed outline) selected for monitoring the MR signal as a function of time. (B) The average MR signals within the ROI as a function of time, normalised against a quadratic baseline, acquired using a RARE and UTE readout.

S4. $T_{1,s}$ and $T_{2,s}$ Effects on NAC Thiol and GSH Amide MTR Profiles

Although $T_{1,s}$ and $T_{2,s}$ relaxation affects saturation efficiency (12), this is not expected to be a significant issue due to the relatively long saturation pulse duration (i.e., seconds) and relatively strong saturation pulse B_1 (i.e., > 1 μT) used in this study. Nevertheless, the effects of various $T_{1,s}$ and $T_{2,s}$ values on the NAC thiol and GSH amide MTR are shown in the BM simulated CD MTR profiles in Figure S6. The BM simulation parameters used were the same as that in the main manuscript, with the exception of $T_{1,s}$ and $T_{2,s}$ being varied. As expected, insignificant changes are observed in the NAC thiol and GSH amide MTR profiles between the different $T_{1,s}$ and $T_{2,s}$ values.

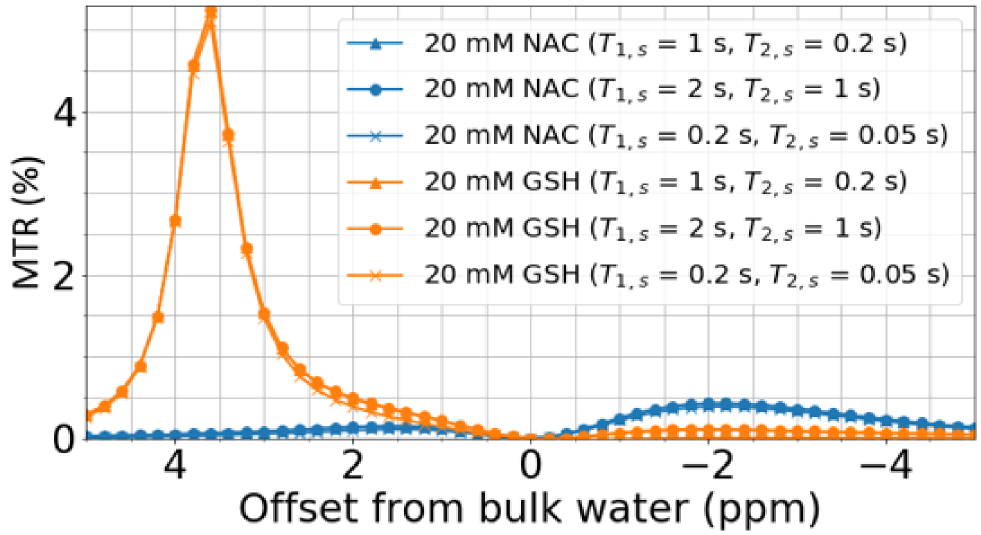

Figure S6 BM simulated CD MTR profiles of 20 mM NAC (blue marked lines) and GSH (orange marked lines) at various $T_{1,s}$ and $T_{2,s}$ values. Some line markers/symbols are overlapped.

S5. Effect of B_0 Drift on MR Signal

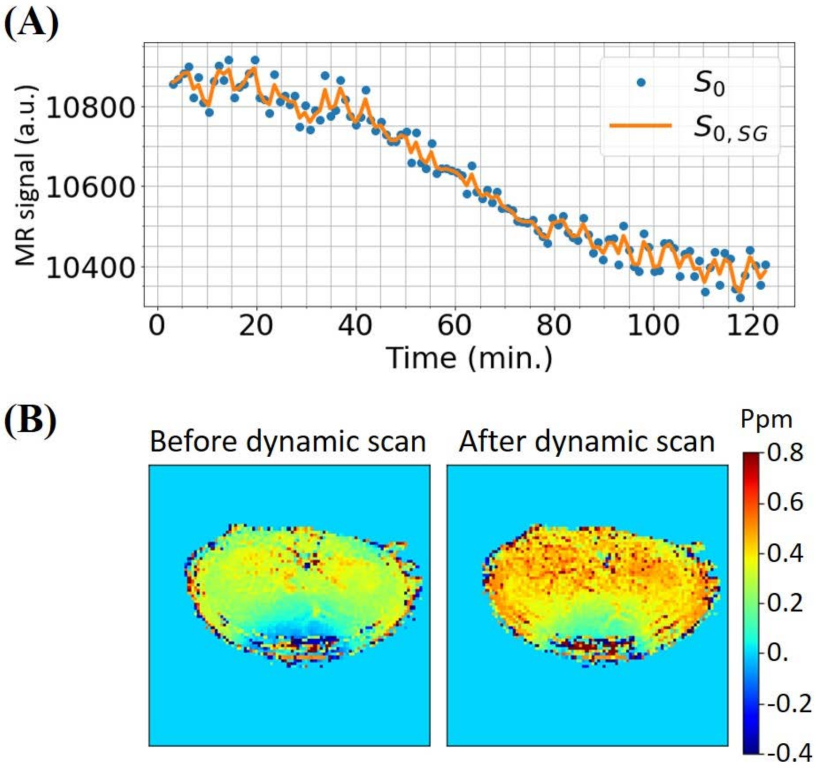

Figure S7 (A) Evidence of S_0 signal drift in the ROI of a representative test mouse, caused by the recurring application of gradient and RF pulses in the CEST-UTE pulse sequence (13,14). The fitted $S_{0,SG}$ baseline was used for normalising the MR signals from the non-S_0 images to compensate for the signal drift. (B) B_0-shift maps of the same mouse acquired before and after the dynamic scan (FL1) to elucidate the B_0 drift phenomenon.

S6. Steady-state Violation in CEST-UTE Experiments

As the CEST-UTE pulse sequence involves k-space acquisitions interleaved with RF saturation pulses, it necessitates an approximate saturation steady-state to be achieved before the dynamic CEST measurement can be made. As the CEST-UTE experiments in this study involves switching the transmitter frequency between different chemical shifts (e.g., 333, -2.7, and 3.6 ppm), the saturation steady-state at any one transmitter frequency is regularly violated. This is illustrated in Figure S8, where the first instance of the signals acquired with ω_{tx} = -2.7 and 3.6 ppm (i.e., FL1) consistently differ from the second instance. Using the same practice as Chen et al. (13), only the second instances were kept for analysis.

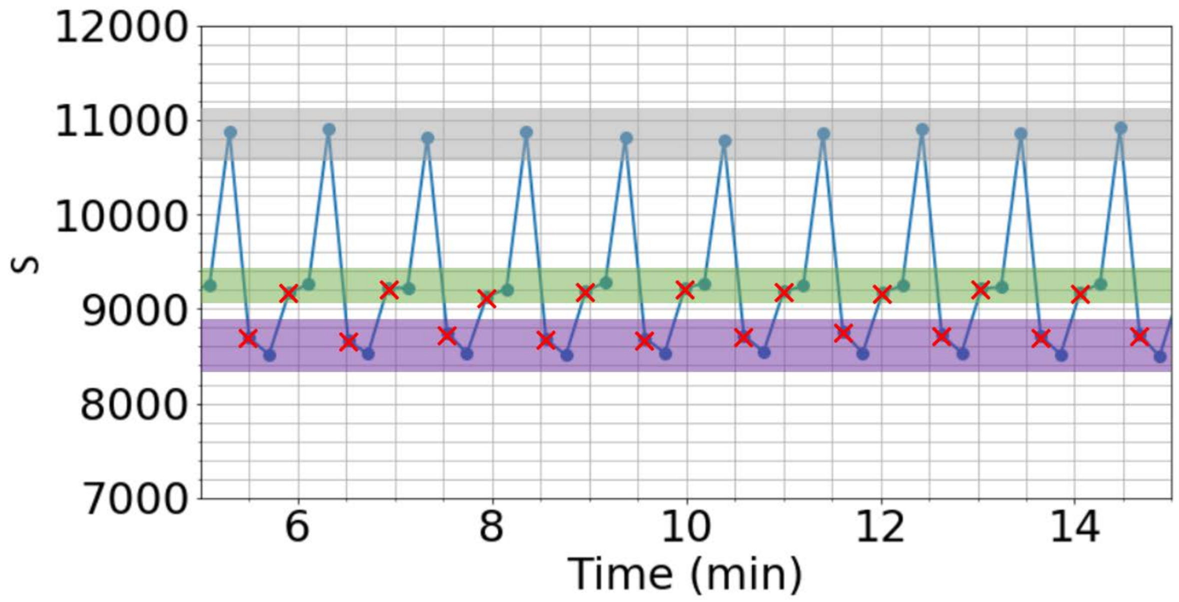

Figure S8 Dynamic MR signal intensities (blue dots) of a representative mouse liver ROI acquired using FL1 in a CEST-UTE experiment. Only part of the dynamic scan (5 – 15 minutes experimental time) is shown. The intensities highlighted in grey, green, and purple represent the MR signals acquired with the saturation transmitter frequency at 333 (i.e., S_0), -2.7, and 3.6 ppm, respectively. The red crosses indicate the removal of the first instance of the doubly-acquired -2.7 and 3.6 ppm signals as they have not reached an approximate steady-state after frequency switching (13). The grey, green, and purple bands indicate the signals acquired when ω_{tx} = 333, 3.6, and 2.7 ppm, respectively.

S7. $T_{1,w}$ and $T_{2,w}$ Effects on NAC Thiol MTR

The effect of $T_{1,w}$ and $T_{2,w}$ values on the NAC thiol MTR were simulated using the 3-pool (NAC thiol, macromolecular-bound water, and free water pools) BM equations to emulate the $T_{1,w}$ and $T_{2,w}$ variations due to variations in paramagnetic species (e.g., iron) concentration. The simulation parameters used include: thiol-water proton exchange rate (k_{sw}) = 6180 s^{-1} (4), bound-free water proton exchange rate (k_{mw}) = 51 s^{-1} (15); chemical shift with respect to water ($\Delta\omega_{sw}$) = −2.7 (NAC thiol) (4) and 0.001 ppm (bound water); M_0 = 0.0028 (NAC thiol) and 0.069 (bound water) (15); NAC thiol T_1 ($T_{1,s}$) = 1 s; NAC thiol T_2 ($T_{2,s}$) = 0.2 s; bound water T_1 ($T_{1,m}$) = 1 s (16); bound water T_2 ($T_{2,m}$) = 74 ms (linearly extrapolated from literature $T_{2,m}$ values at B_0 = 1.5 and 3 T (15)); water T_1 ($T_{1,w}$) = 995 ms (5); water T_2 ($T_{2,w}$) = 23 ms (5). The parameters for a train of Gaussian saturation pulses (max B_1 = 1.2 μT, pulse duration = 30 ms, inter-pulse delay = 10.4 ms, number of pulses = 302) was used in the simulation to emulate the CEST-UTE saturation scheme.

As shown in Figure S9, the NAC thiol MTR increases with $T_{1,w}$ and $T_{2,w}$, which is consistent with theoretical CEST models (12). Interestingly, the NAC thiol MTR increases almost linearly with $T_{1,w}$ between 0.5 – 2.0 s but increases non-linearly with $T_{2,w}$ between 0.001 – 1 s. Increasing $T_{2,w}$ beyond the literature value of 0.023 s produces little increase in the NAC thiol MTR.

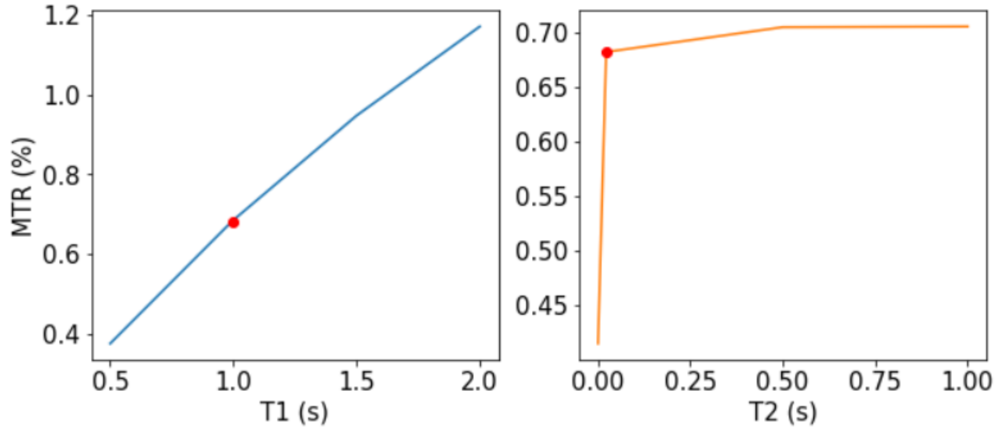

Figure S9 Simulated NAC thiol MTR values at different $T_{1,w}$ and $T_{2,w}$ values. The red dots indicate the literature mouse liver T_1 (0.995 s) and T_2 (0.023 s) values at B_0 = 7 T, as used in other simulations in this study.

S8. Hepatic NAC Concentration from ^{1}H-NMR Spectral Deconvolution

The hepatic NAC concentration was determined from the ^{1}H-NMR spectra of aqueous liver extracts from the test mice group by using the software, Newton (17), to deconvolute the NAC methyl proton peak (Figure 5 in main manuscript) and the reference TSP-d_4 methyl proton

peak. For each 1H-NMR spectrum, hepatic NAC concentration was determined using the equation

$$Hepatic\ [NAC] = \left(\frac{3 \times Int_{NAC}}{Int_{TSP}} \times [TSP]\right) \times \frac{V_1 \times d_{liver}}{m_{liver}},$$

where Int_{NAC} is the integral value of the NAC methyl proton peak determined from the spectral devolution, Int_{TSP} is the integral value of the TSP-d_4 methyl proton peak determined from the spectral devolution, [TSP] = 0.71 mM, V_1 = 0.00058 L is the buffer solution volume used to resuspend the aqueous liver extracts, and d_{liver} = 1000 g/L (18) and m_{liver} are the literature density and mass of the mice liver, respectively.

The average hepatic NAC concentration was determined to be 0.12 ± 0.03 mM at 8 minutes post-injection.

References

1. Chan KWY, McMahon MT, Kato Y, Liu G, Bulte JWM, Bhujwalla ZM, Artemov D, van Zijl PCM. Natural D-Glucose as a Biodegradable MRI Contrast Agent for Detecting Cancer. Magn Reson Med 2012;68(6):1764-1773.
2. Xu X, Yadav NN, Song X, McMahon MT, Jerschow A, van Zijl PCM, Xu J. Screening CEST Contrast Agents Using Ultrafast CEST Imaging. J Magn Reson 2016;265:224-229.
3. Hennig J, Nauerth A, Friedburg H. RARE Imaging: A Fast Imaging Method for Clinical MR. Magn Reson Med 1986;3(6):823-833.
4. Chen J, Yadav NN, Stait-Gardner T, Gupta A, Price WS, Zheng G. Thiol-Water Proton Exchange of Glutathione, Cysteine, and N-Acetylcysteine: Implications for CEST MRI. NMR Biomed 2020;33(1):e4188.
5. Chow AM, Gao DS, Fan SJ, Qiao Z, Lee FY, Yang J, Man K, Wu EX. Measurement of Liver T_1 and T_2 Relaxation Times in an Experimental Mouse Model of Liver Fibrosis. J Magn Reson Imaging 2012;36(1):152-158.
6. van Zijl PCM, Lam WW, Xu J, Knutsson L, Stanisz GJ. Magnetization Transfer Contrast and Chemical Exchange Saturation Transfer MRI. Features and Analysis of the Field-Dependent Saturation Spectrum. NeuroImage 2018;168:222-241.
7. Zaitsev M, Maclaren J, Herbst M. Motion Artifacts in MRI: A Complex Problem with Many Partial Solutions. J Magn Reson Imaging 2015;42(4):887-901.
8. Glover GH, Pauly JM. Projection Reconstruction Techniques for Reduction of Motion Effects in MRI. Magn Reson Med 1992;28(2):275-289.
9. Kim B, So S, Park H. Optimization of Steady-State Pulsed CEST Imaging for Amide Proton Transfer at 3 T MRI. Magn Reson Med 2019;81(6):3616-3627.
10. Khlebnikov V, Geades N, Klomp DWJ, Hoogduin H, Gowland P, Mougin O. Comparison of Pulsed Three-Dimensional CEST Acquisition Schemes at 7 Tesla: Steady State Versus Pseudosteady State. Magn Reson Med 2017;77(6):2280-2287.
11. Sun PZ, Benner T, Kumar A, Sorensen AG. Investigation of Optimizing and Translating pH-Sensitive Pulsed-Chemical Exchange Saturation Transfer (CEST) Imaging to a 3T Clinical Scanner. Magn Reson Med 2008;60(4):834-841.
12. Zhou J, Wilson DA, Sun PZ, Klaus JA, van Zijl PCM. Quantitative Description of Proton Exchange Processes Between Water and Endogenous and Exogenous Agents for WEX, CEST, and APT Experiments. Magn Reson Med 2004;51(5):945-952.

13. Chen L, Wei Z, Chan KWY, Cai S, Liu G, Lu H, Wong PC, van Zijl PCM, Li T, Xu J. Protein Aggregation Linked to Alzheimer's Disease Revealed by Saturation Transfer MRI. NeuroImage 2019;188:380-390.
14. Smith AM, Lewis BK, Ruttimann UE, Ye FQ, Sinnwell TM, Yang Y, Duyn JH, Frank JA. Investigation of Low Frequency Drift in fMRI Signal. NeuroImage 1999;9(5):526-533.
15. Stanisz GJ, Odrobina EE, Pun J, Escaravage M, Graham SJ, Bronskill MJ, Henkelman RM. T1, T2 Relaxation and Magnetization Transfer in Tissue at 3T. Magn Reson Med 2005;54(3):507-512.
16. Tunnicliffe EM, Banerjee R, Pavlides M, Neubauer S, Robson MD. A Model for Hepatic Fibrosis: The Competing Effects of Cell Loss and Iron on Shortened Modified Look-Locker Inversion RecoveryT1(shMOLLI-T1) in the Liver. J Magn Reson Imaging 2017;45(2):450-462.
17. Chylla RA, Hu K, Ellinger JJ, Markley JL. Deconvolution of Two-Dimensional NMR Spectra by Fast Maximum Likelihood Reconstruction: Application to Quantitative Metabolomics. Anal Chem 2011;83(12):4871-4880.
18. Miller WH, Hartmann-Siantar C, Fisher D, Descalle M-A, Daly T, Lehmann J, Lewis MR, Hoffman T, Smith J, Situ PD, Volkert WA. Evaluation of Beta-Absorbed Fractions in a Mouse Model for 90Y, 188Re, 166Ho, 149Pm, 64Cu, and 177Lu Radionuclides. Cancer Biotherapy and Radiopharmaceuticals 2005;20(4):436-449.
